# Tumor-on-a-chip models combined with mini-tissues or organoids for engineering tumor tissues

**DOI:** 10.7150/thno.90093

**Published:** 2024-01-01

**Authors:** Hanjun Hwangbo, SooJung Chae, Wonjin Kim, Seoyul Jo, Geun Hyung Kim

**Affiliations:** Department of Precision Medicine, Sungkyunkwan University School of Medicine (SKKU-SOM) Suwon 16419, Republic of Korea

**Keywords:** Tumor-on-a-chip, Tumor microenvironment, Organoids, Biofabrication, Cancer research

## Abstract

The integration of tumor-on-a-chip technology with mini-tissues or organoids has emerged as a powerful approach in cancer research and drug development. This review provides an extensive examination of the diverse biofabrication methods employed to create mini-tissues, including 3D bioprinting, spheroids, microfluidic systems, and self-assembly techniques using cell-laden hydrogels. Furthermore, it explores various approaches for fabricating organ-on-a-chip platforms. This paper highlights the synergistic potential of combining these technologies to create tumor-on-a-chip models that mimic the complex tumor microenvironment and offer unique insights into cancer biology and therapeutic responses.

## Introduction

Cancer remains one of the toughest challenges in modern medicine, affecting millions of people worldwide and presenting complex, multifaceted obstacles for researchers and clinicians alike 1. Traditional two-dimensional (2D) cell cultures have been the backbone of cancer research for decades; however, they often fail to capture the intricacies and heterogeneity of tumors *in vivo* 2. Consequently, there is a growing demand for more sophisticated and physiologically relevant models to study cancer biology, drug responses, and potential therapeutic interventions 2, 3.

In recent years, rapid advancements in biofabrication techniques have led to a new era in tissue engineering and regenerative medicine. These innovative methods have enabled the construction of three-dimensional (3D) mini-tissues and organoids that better mimic the structural and functional complexities of native tissues and organs 4-8. By incorporating these cutting-edge biofabrication approaches, researchers have made significant steps in developing advanced *in vitro* models known as “organ-on-a-chip” systems 9.

Researchers have utilized various biofabrication methods such as 3D bioprinting 10, 11, spheroids 12, microfluidic systems 13, and self-assembly techniques 14 using cell-laden hydrogels to recapitulate tissue-like microenvironments. For instance, the introduction of cell-based components that imitate key features of human tumors and the surrounding microenvironment has allowed close representations of tumor microenvironments 9, 15, 16.

In the following sections, we delve into a range of biofabrication techniques used to fabricate mini-tissues. Here, we discuss both the strengths and limitations of each of the approaches. These methods encompass 3D Bioprinting, which offers precise control over the spatial arrangement of cells and biomaterials, enabling the creation of intricate structures 10, 11. Furthermore, spheroid formation which leverages cell self-assembly to produce mini-tissues, providing a unique approach to tissue engineering 12, 14. Additionally, we examine microfluidic systems, which have the capability to generate complex cellular structures. Lastly, self-assembly techniques rely on the inherent properties of cell-laden hydrogels, facilitating the formation of three-dimensional structures 13. These techniques collectively offer a diverse toolkit for engineering mini-tissues, each with its own set of advantages and considerations.

Subsequently, we will explore the various techniques employed in fabricating organ-on-a-chip platforms. These platforms offer dynamic and controlled microenvironments that enable researchers to mimic the essential functions of specific organs, making them powerful tools for studying normal physiology and disease progression. Additionally, we will examine microfluidics-based organ-on-a-chip models, biomaterial-based approaches employing biologically relevant scaffolds, and hybrid systems that integrate both technologies 13, 17.

Building on this foundation, we will investigate the exciting area of tumor-on-a-chip models, where mini-tissues or organoids are merged with advanced microfluidic systems to create physiologically relevant tumor microenvironments (TMEs). These innovative platforms can provide new insights into cancer biology, including tumor heterogeneity, invasion, metastasis, and responses to therapeutic agents 18, 19. By examining various tumor-on-a-chip models, we demonstrate the unique advantages of each biofabrication method and their potential applications in the context of cancer research.

This review aims to shed light on promising developments in the integration of tumor-on-a-chip technology with mini-tissues or organoids through diverse biofabrication methods. The synergy between these cutting-edge technologies can bridge the gap between traditional 2D cell culture and *in vivo* studies, providing researchers with more reliable and representative *in vitro* models to explore the complexities of cancer biology and therapeutic interventions. Through an improved understanding of tumor behavior and drug responses, these advanced models may pave the way for personalized medical approaches and propel us closer to more effective and targeted cancer treatments.

## Biofabrication methods for mini-tissues/organoids

Mini-tissues, also known as organoids or tissue-like structures, have attracted considerable attention in recent years. They offer a more physiologically relevant and intricate representation of native tissues than traditional 2D cell cultures. Biofabrication methods employed to construct mini-tissues utilize various innovative techniques, each presenting unique advantages and challenges in generating tissue-like structures with cellular complexity and functionality. In this section, we examine several biofabrication methods in detail and discuss their principles, applications, and contributions to cancer research.

### 3D Bioprinting

3D bioprinting is a revolutionary biofabrication technique that allows the precise control of the spatial arrangement of cells, biomaterials, and biochemical cues, leading to the creation of complex multicellular structures 5, 20-22. This method uses bioinks, which are cell-laden hydrogels or biomaterials, as building blocks that can be patterned layer-by-layer to form 3D tissue-like structures 23. The ability to create tissues with intricate architectures and controlled cell distributions makes 3D bioprinting a valuable tool for mini-tissue construction.

Various biocompatible and biodegradable materials have been applied as bioinks in 3D bioprinting. In particular, hydrogels such as alginate, gelatin, collagen, and fibrin are commonly used because of their biochemical and biophysical abilities to mimic the extracellular matrix (ECM) and support cell growth and differentiation 24-26. In addition, decellularized ECM-based bioinks derived from porcine, bovine, and fish tissues better simulate native microenvironments, facilitating the interaction and organization of cells 27, 28. Various examples of 3D bioprinting processes used to obtain functional 3D mini-tissue constructs are listed in Table 1.

**Figure 1:**
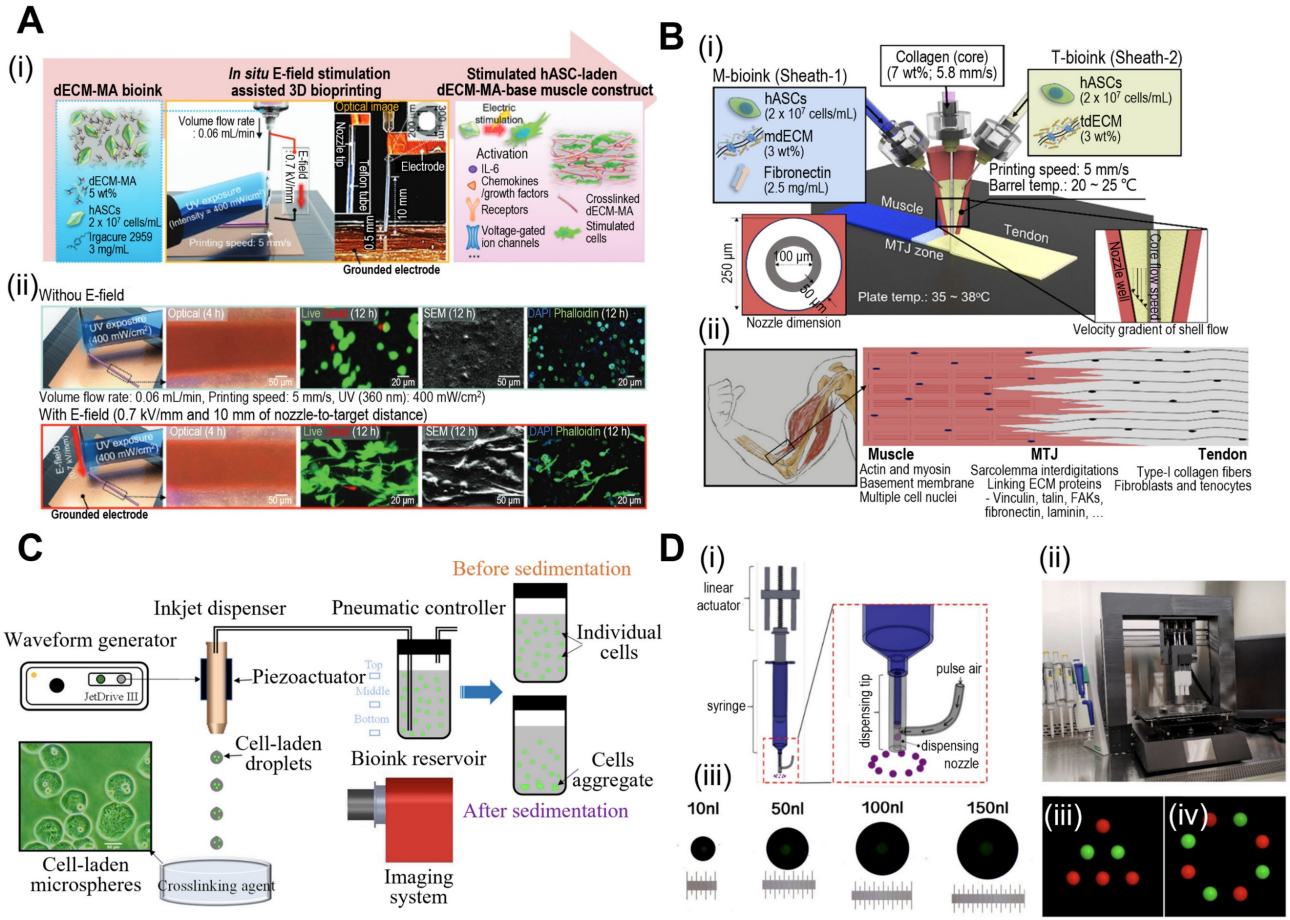
**Fabrication of mini-tissues/organoid using bioprinting system.** (**A**) (i) Schematic diagram of *in situ* E-field stimulation and (ii) optical, live (green)/dead (red), SEM, and DAPI (blue)/phalloidin (green) images of hASCs fabricated W/ or W/O E-fields, where *in situ* E-field simulation evoked favourable cellular responses. Adapted with permission from 29, copyright Wiley 2021. (**B**) Schematic illustration of (i) the fabrication process of 3D bioprinting using a modified core/shell nozzle consisting of two sheath inlets and, and (ii) myotendinous junction (MTJ) unit. Adapted with permission from 31, copyright Wiley 2022. (**C**) Schematic illustration of inkjet bioprinting system containing several components including piezoactuator-attached inkjet nozzle, a waveform generator, a pneumatic controller, a bioink reservoir. Adapted with permission from 32, copyright MDPI 2022. (**D**) (i) Schematical diagram and (ii) optical images of drop-on-demand bioprinting method. (iii) Representative images of various patterns of dispensed solution volume by the drop-on-demand printing system. Adapted with permission from 33, copyright MDPI 2020.

In 3D bioprinting, various printing techniques, including extrusion, inkjet, and laser-assisted methods are employed. Extrusion-based bioprinting utilizes a syringe or nozzle to deposit the bioink layer-by-layer, allowing precise control over cell and material placement. Furthermore, extrusion-based bioprinting can be enhanced through functionalization of the extrusion process. Kim et al. demonstrated simultaneous *in situ* E-field stimulation during bioink extrusion, as shown in Fig. 1A 29. After 12 h of stimulation, cells developed branched actin filaments. E-field stimulation results in favorable cellular responses, such as increased cell proliferation, alignment, and myogenic activity in human adipose stem cells (hASCs). In addition, core-shell nozzles are commonly employed in tissue engineering applications to create hierarchical structures 30. This nozzle application could be further expanded by incorporating double-sheath channels, as illustrated in Fig. 1B 31. When this nozzle was used in extrusion-based bioprinting, researchers successfully developed a muscle-tendon unit with significant upregulation of genes associated with the muscle-tendon junction.

Inkjet-based bioprinting employs thermal or piezoelectric methods to eject bioink droplets from a specified location. In contrast, laser-assisted bioprinting uses lasers to propel bioink droplets to the target area. Consequently, this is considered a drop-on-demand (DOD) bioprinting technique, and recent advances suggest its great promise for fabricating functional tissues for implants and drug development. Xu et al. investigated the piezoelectric-actuated bioprinting of cell-bearing alginate bioink directly into a crosslinking agent to develop cell-laden microspheres (Fig. 1C) 32. However, the current forms of DOD bioprinting are limited by inconsistent droplet volumes and negative effects on cell viability after dispensing at high pressures. To address these limitations, Grottkau et al. developed direct volumetric DOD bioprinting as described in Fig. 1D 33. The described bioprinter utilizes linear actuator-driven syringes that can dispense <10 nL with high accuracy (±5%).

In recent years, digital light-processing (DLP)-based bioprinting methods have revolutionized the creation of mini-tissues with intricate architectures 34. This technique employs concentrated light to solidify photo-crosslinkable solutions, resulting in the formation of 3D mini-tissues 35. The method offers several advantages, including rapid production, flexibility, and high resolution, making DLP-based bioprinted mini-tissues highly desirable. Xie et al. developed a DLP-based bioprinting system to create osteo-callus organoids composed of microspheres loaded with BMSCs 36. After 4 weeks of implantation, the organoid displayed significant new bone formation. Additionally, Carberry et al. crafted PEG-based sacrificial models with intricate architectures (37 ± 4 μm) 37. These molds were cast into Matrigel to transfer arrays of features. The authors affirmed that utilizing DLP-printed sacrificial molds offers a robust and rapid method to obtain 3D mini-tissues.

From the perspective of cancer research, 3D bioprinted mini-tissues provide valuable insights into tumor growth, invasion, and drug responses. By incorporating different cell types, including several stem cells and ECM components, researchers can create tumorlike structures that closely mimic the TME, allowing the study of tumor-stromal interactions, angiogenesis, and immune cell infiltration 38, 39. Among various types of tumor models, the 3D bioprinting of glioblastoma tumors has been well studied, as it is an aggressive form of cancer that affects the central nervous system 40. For instance, Dai et al. fabricated glioma stem cell-laden bioconstructs composed of gelatin, alginate, and fibrinogen 41. The cells proliferated well and showed high differentiation potential (glial fibrillary acidic protein and β-tubulin III). Moreover, the 3D tumor model showed higher drug resistance to temozolomide than the 2D model. Additionally, inkjet bioprinting was used to obtain copatterned hepatoma and glioma constructs to evaluate the efficacy of chemotherapeutics (in this case tegafur) against cells 42. Researchers have concluded that the developed approach allows precise patterning (at the microscale) of various cells onto microchips for accurate analysis of drug efficacy. In addition, Wang et al. developed a 3D mini-tissue composed of human lung cancer cells (A549/95-D) using a 3D bioprinting process 43. The cancer cells were combined with a gelatin-alginate-based bioink and extruded to form 3D constructs, which were subsequently crosslinked in a sodium alginate solution. To investigate cancer invasion, the authors assessed matrix metalloproteinases 2 (MMP2) and matrix metalloproteinases 9 (MMP9) using qPCR. The results revealed a significant upregulation of these genes in cells cultured within 3D constructs compared to those in 2D culture. Based on these examples, 3D bioprinting can enable the creation of patient-specific tumor models, paving the way for personalized medical approaches. However, the absence of adequate cell-cell interactions is a significant limitation in the realm of 3D bioprinted tissues. Research has demonstrated that robust cell-cell interactions, which affect intricate intracellular signaling, can substantially enhance the bioactivities of the cells within a tissue. To address this concern, researchers have explored cellular aggregates called cell spheroids, which can bolster the lacking cell-cell interactions in 3D bioprinted tissues. The subsequent sections delve into the fabrication and applications of cell-spheroids as mini-tissues.

### Spheroids

Spheroids are self-assembled cellular aggregates that recapitulate certain aspects of tissue architecture and function 44. These unique 3D spherical structures are formed through cell-cell interactions and exhibit oxygen, nutrient, and signaling molecule gradients, resulting in physiologically relevant tissue models 12. Before the introduction of spheroids by Moscona and Moscona 45, 2D cell cultures were used for *in vitro* drug testing, disease modeling, and cellular response evaluations. Although 2D cell cultures are inexpensive and reproducible, their 2D characteristics do not reflect those of the 3D *in vivo* microenvironment. Thus, cellular spheroids can amend the limitations of 2D characteristics by providing extensive cell-to-cell interactions and mimicking the regulation of *in vivo*-like cellular functions including cell proliferation, migration, and differentiation 46, 47.

**Figure 2:**
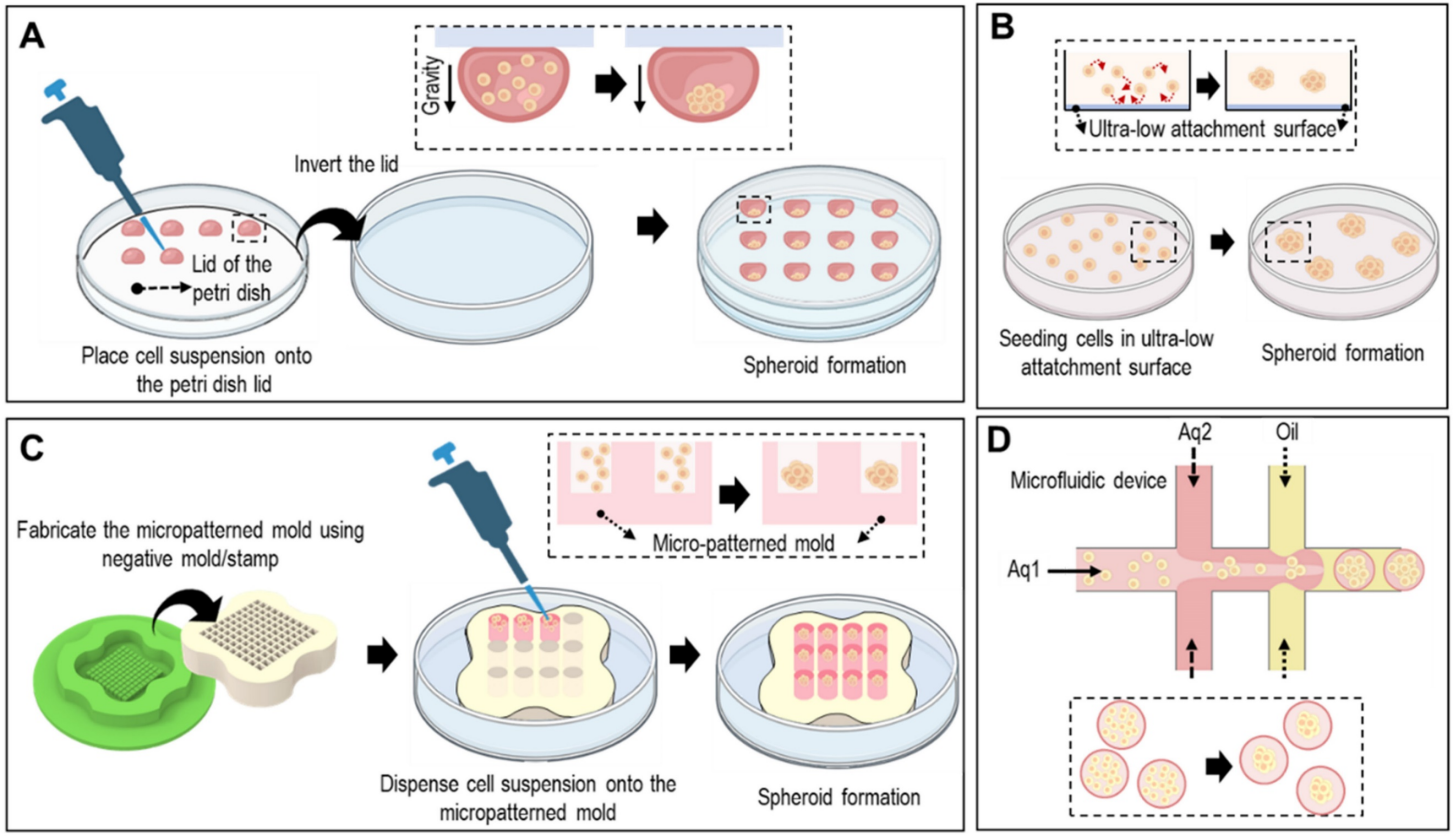
**Various fabrication methods of cell-spheroids.** Various schematical illustration of spheroid formations including (**A**) hanging drop that utilizes gravitational forces and surface tension to induce cell aggregation, (**B**) non-adherent surface which applies low attachment surfaces to induce self-assembly of cells into spheroids, (**C**) micro-patterned mold which utilizes seeding cells into micropatterned mold, and (**D**) microfluidic method which utilizes immiscible fluids and precise control of the flowrates to generate spheroids.

Spheroids can be formed using various methods, including hanging drop techniques, ultra-low-attachment plates, micropatterned molds, and microfluidic devices (Fig. 2). In the hanging-drop method, cell suspensions are pipetted onto the inverted lid of a culture dish, allowing the cells to self-assemble into spheroids through gravity-driven aggregation, as shown in Fig. 2A. Ultra-low attachment plates prevented cell attachment to the surface, promoting spheroid formation by facilitating cell-cell interactions (Fig. 2B). As shown in Fig. 2C, micropatterned molds with non-adhesive surfaces can be utilized to form spheroids. Briefly, seeded cells aggregate *via* the gravitational force. However, controlling spheroid size and handling is difficult. To overcome these limitations, microfluidic devices offer precise control over cell-seeding and culture conditions, enabling the generation of uniform spheroids of defined sizes (Fig. 2D).

**Figure 3:**
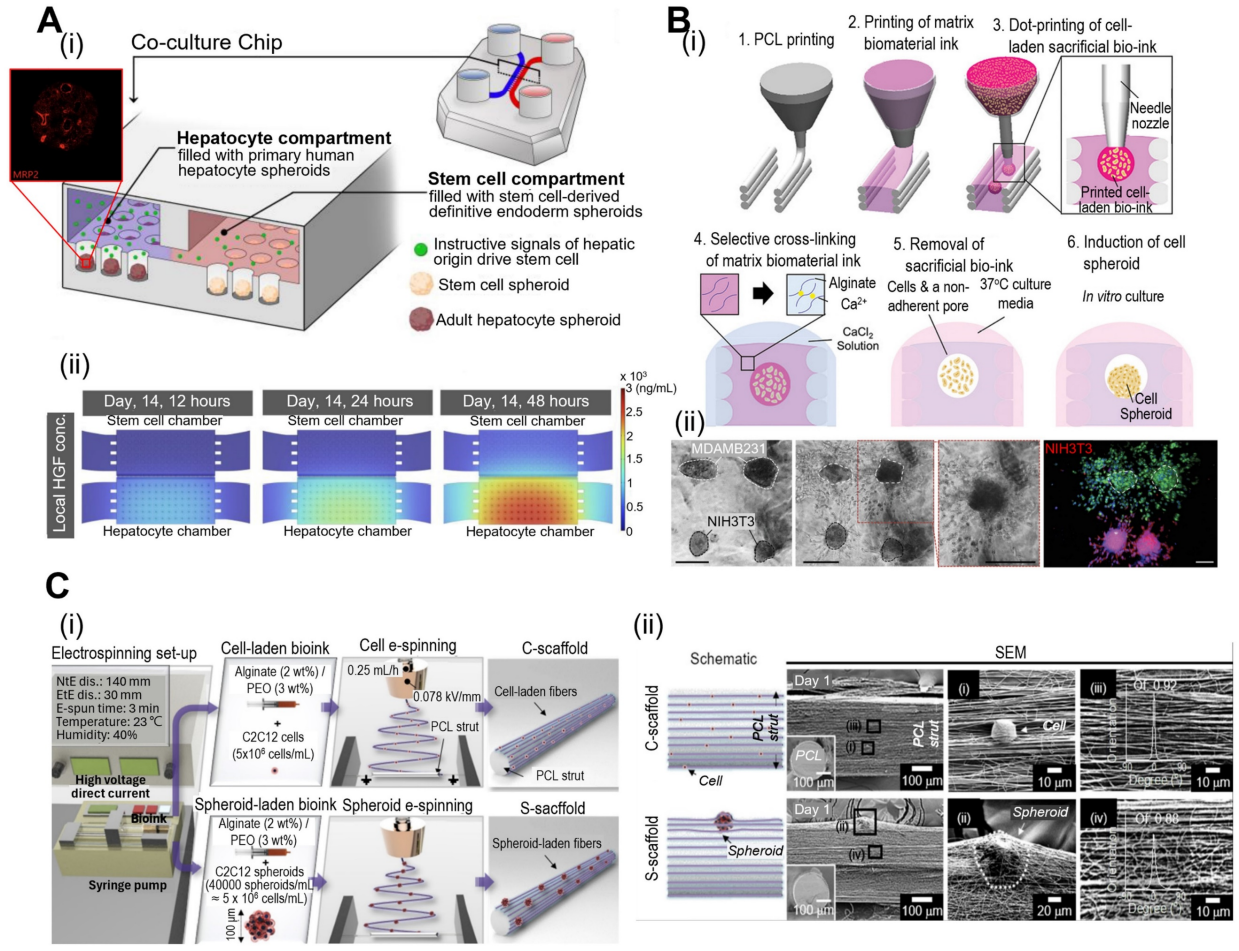
**Fabrication of mini-tissues/organoid using cell-spheroids.** (**A**) (i) Schematic diagrams of the co-culture microfluidic device and (ii) computational fluid dynamic model of the heatmaps of) demonstrating rapid accumulation HGF (hepatocyte growth factor) using COMSOL. Adapted with permission from 48, copyright MDPI 2023. (**B**) (i) “Bio-dot printing” fabrication procedure using polycaprolactone printing and cell spheroids, and (ii) Microscopy and fluorescence images of MDAMB231 and/or NIH3T3 cell spheroids demonstrating 3D spheroid invasion. Adapted with permission from 49 copyright Wiley 2020. (**C**) (i) Schematic diagram of cell/spheroid electrospinning process and (ii) scanning electron microscopy images demonstrating embedded cells (C-scaffold) and cell spheroid (S-scaffold) in electrospun PCL nanofibers. Adapted with permission from 50 copyright Ivyspring 2021.

Spheroids offer several advantages such as ease of formation, low cost, and the ability to study multicellular interactions. Moreover, cell spheroids can exhibit a high expression of tissue-specific genes, thus regulating cell signaling and cytokine expression. For example, Fattahi et al. developed a microfluidic coculture device with two compartments containing human hepatocytes or human pluripotent stem cells (Fig. 3A) 48. The compartments have interconnected grooves that allow for the exchange of paracrine signals. As a result, stem cells differentiate along the hepatic lineages owing to the release of hepatocyte growth factors in compartments containing human hepatocyte spheroids. However, cell spheroids also have limitations, such as limited size control and difficulties in vascularization, which can restrict their application in mimicking larger and more complex tissues.

To overcome these issues, cell spheroids have been incorporated into 3D bioconstructs to significantly enhance their bioactivity. Jeon et al. have developed a “3D Bio-Dot Printing” system that allows precise positioning of multi-type cell spheroids (Fig. 3B) 49. As shown in Fig. 3B(i), this process involved sequential polycaprolactone (PCL) mold printing, biomaterial-based hydrogel printing, and bioink deposition. Consequently, the hydrostatic forces of the hydrogel induced cell aggregation and the formation of cell spheroids [Fig. 3B(ii)]. Fig. 3C shows the incorporation of myoblast (C2C12) spheroids into micro/nanofibrous structures *via* spheroid electrospinning 50. Briefly, a C2C12 spheroid (~100 μm) bearing a bioink consisting of alginate (2 wt%) and PEO (3 wt%) was electrospun into a 3D bundle structure. Owing to the synergistic effects of appropriate topographical cues of micro/nanofibers and the strong cell-to-cell interactions of cell spheroids, myogenic activities were significantly upregulated compared to conventional structures. In addition to these spheroid applications, further advantages and disadvantages of various spheroid applications are explained in Table 1. As demonstrated by the examples provided, cell spheroids have shown the capacity to stimulate robust cell-cell interactions, ultimately enhancing cellular bioactivities. Nonetheless, there are limitations associated with the precise control of cell spheroid size and the extended duration required for their formation. To address these challenges, there have been developments in microfluidic systems for the construction of mini-tissues. These systems enable a rapid fabrication process with precise control over geometries. In the subsequent section, we will delve into the process and applications of microfluidic-based mini-tissue formations.

### Microfluidic systems

Microfluidic systems have emerged as powerful tools for creating mini-tissues owing to their ability to control fluid flow, cell seeding, and culture conditions in microscale environments. Microfluidic devices are typically manufactured from biocompatible materials, such as poly(dimethyl siloxane) (PDMS), poly(methyl methacrylate) (PMMA), and cyclic olefin polymer (COP). In this context, silicone-based elastomer (PDMS) is the most commonly used material owing to its diverse characteristics, including optical transparency, low cost, easy fabrication of intricate structures, bioinertness, and gas permeability 51, 52. Similarly, PMMA has been extensively studied for use in microfluidic devices using various methods including milling, hot embossing, micromachining, laser ablation, and microinjection molding 53. Furthermore, PMMA is a notably more rigid polymer compared to PDMS, rendering it more suitable for mass production 54, 55. However, owing to its rigidity, PMMA is not suitable for valve applications in microfluidic devices. In addition, COPs have demonstrated high resistance to both chemical and biological factors while maintaining optical transparency 56-58. This makes them an excellent choice of polymer for microfluidic applications. Various fabrication methods, such as laser ablation, micromilling, injection molding, hot embossing, and nanoimprint lithography, can be employed in the production of microfluidic devices. The chips were designed to accommodate multiple channels, reservoirs, and chambers to regulate fluid flow and cell culture. These platforms enable the generation of precise tissue architectures and dynamic microenvironments 13, 59.

**Figure 4:**
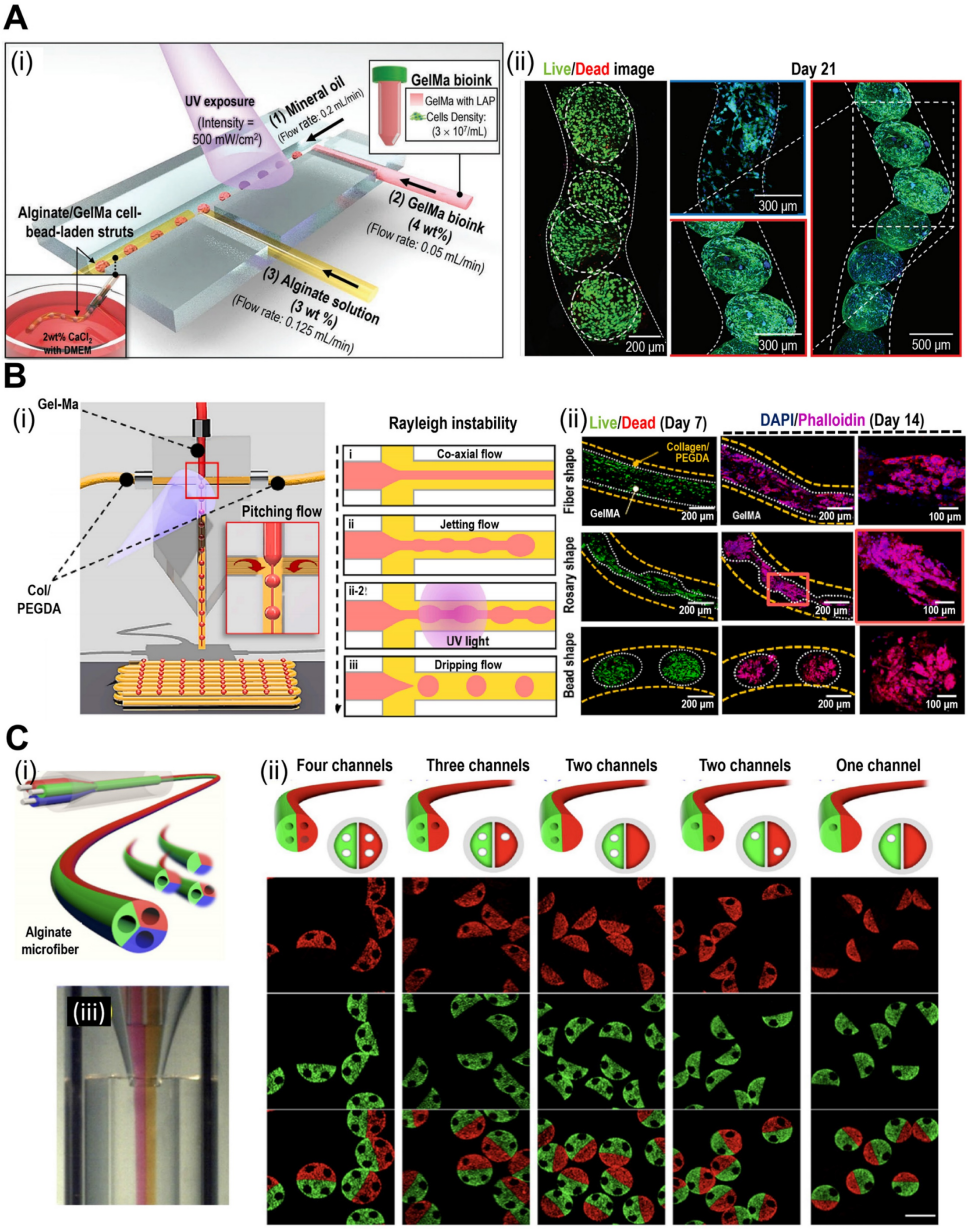
**Fabrication of mini-tissues/organoid using microfluidic system.** (**A**) (i) Schematic diagrams, and (ii) live (green)/dead (red) and DAPI (blue)/phalloidin (green) images demonstrating cell-bead laden structures fabricated with the microfluidic device. Adapted with permission from 60, copyright Wiley 2022. (**B**) (i) Schematic diagram of the T-junction microfluidic system and the influence of the Rayleigh instability of cell-laden methacrylated gelatin (Gel-Ma) and collagen/polyethylene glycol diacrylate (PEGDA). (ii) Live (green)/dead (red) and DAPI (blue)/phalloidin (red) images of cells in fiber, rosary, and bead core structures. Adapted with permission from 61, copyright Elsevier 2023. (**C**) Schematic diagram of various geometries of microchannels (i-ii) and (iii) optical images of the bioink flowing process. Adapted with permission from 62, copyright Elsevier 2021.

Microfluidic systems are commonly used to obtain spheroid-like structures. As shown in Fig. 4A(i), the microfluidic device can be used to aggregate cells into a bead structure 60. Based on careful consideration of the flow rates in each channel, cell beads with sizes that enhance cell-to-cell interactions were obtained, as shown in Fig. 4A(ii). This approach can be further enhanced by introducing two side channels that provide a pitching flow [Fig. 4B(i)] 61. By controlling the side channel flow rate, various core structures, including beads, rosaries, and fibers, were obtained, as demonstrated in the live (green)/dead (red) and DAPI (blue)/phalloidin (red) images shown in Fig. 4B(ii).

Microfluidic systems enable the controlled seeding of cells and the establishment of tissue-like structures within defined microenvironments. By adjusting the flow rates and culture conditions, researchers can create gradients of nutrients, oxygen, and other factors to mimic the *in vivo* microenvironment. Chai et al. fabricated a microgel structure consisting of a core (collagen type I) and a shell (alginate) using a multichannel microfluidic device 62. Cell-laden microgels were incorporated into methacrylated silk fibroin (Sil-Ma) and methacrylated gelatin (Gel-Ma) hydrogels for bioprinting. As a result of microgel incorporation, cellular proliferation significantly improved. Cheng et al. fabricated microfibers using a multibarrel capillary microfluidic device [Fig. 4C(ii)] 63. The described system can be further elaborated *via* the incorporation of several microchannels, thereby obtaining fibers with a variety of microarchitectures. Microfluidic systems offer excellent reproducibility and scalability, making them ideal for high-throughput studies and drug-screening applications. The ability to generate mini-tissues in a controlled and standardized manner enhances the reliability of experimental results. Microfluidic-based fabrication methods, while advantageous, come with certain drawbacks. These include the complex design and fabrication process, restricted selection of biomaterials, and careful management of cell viability due to potential shear stress in microfluidic environments. Consequently, researchers have sought to address these challenges by integrating various fabrication methods into hybrid approaches. These hybrid methods offer potential solutions to the limitations posed by individual techniques. In the subsequent section, we will explore the applications of hybrid fabrication methods in the creation of mini-tissues.

### Hybrid fabrication methods using cell-laden hydrogels

Hybrid fabrication techniques in tissue engineering capitalize on the unique properties of cell-laden hydrogels. These hydrogels, which contain living cells, provide an ideal environment for cellular growth and tissue formation. By combining various methods, these techniques enable the creation of intricate three-dimensional structures that closely resemble natural tissues. This versatility allows for the generation of mini-tissues with crucial cell-cell interactions, along with tissue-specific functionalities and microarchitectures. This level of precision holds significant promise for advancing the field of tissue engineering as well as tumor development and therapeutics assessments. For instance, microfluidic-assisted bioprinting of intricate structures has been reported 64. Costantini et al. utilized a three-channeled microfluidic bioprinting system consisting of the cell-bearing bioink in the core channel, and the crosslinker-bearing hydrogel in the side channel which formed a pitching flow to the core 65.

Consequently, the diameter of the core fiber can be reduced and crosslinked simultaneously. Similarly, Dickman et al. utilized a microfluidic chip with a calcium chloride solution in the side channels to crosslink the alginate hydrogel in the core simultaneously during the extrusion process. Consequently, the printability of the proposed system was significantly increased 66.

Challenges such as inadequate vascularization and limited size can impede the mimicking of larger and more complex tissues in cell spheroids. To address this issue, cell spheroids are incorporated into 3D bioprinted structures. Kim et al. developed a new bioprinting system that utilizes bioink droplets to obtain interlayered human umbilical vein endothelial cell (HUVEC) spheroids within 3D bioconstructs containing hASCs 67. DAPI (blue)/phalloidin (red) and DAPI (blue)/OPN (green) images indicate that F-actin was significantly more developed with the incorporation of cell spheroids, and the osteogenic activities of hASCs were elevated compared to conventionally obtained bioconstructs. Similarly, osteogenesis-related genes (BMP-2, ALP, OCN, and OPN) and angiogenesis-related genes (VEGF, PECAM1, and VWF) were significantly upregulated in the described constructs.

Mini-tissue assembled using hybrid fabrication processes holds great promise for tissue engineering and cancer research. By modulating the hydrogel properties and incorporating different cell types, researchers can create tissue-specific models. In tissue engineering, upon the implantation of tissue-specific bioconstructs, tissue integration with native tissue can be accelerated, resulting in functional recovery. Furthermore, tissue-specific models can be developed to recreate the TME for studying tumor development, invasion, and therapeutic responses.

Diverse biofabrication methods for mini-tissues offer unique capabilities to generate tissue-like structures with cellular complexity and physiological relevance. By connecting the potential of 3D bioprinting, spheroids, microfluidic systems, and hybrid fabrication approaches, researchers have paved the way for more advanced and sophisticated tumor-on-a-chip models 40, 68-70. In the subsequent sections, we explore the integration of these mini-tissues with organ-on-a-chip (OOC) platforms to create comprehensive tumor-on-a-chip models, offering unprecedented opportunities to unravel the complexities of cancer biology and advance personalized medicine.

## Fabrication techniques for OOCs

OOC technology has revolutionized biomedical research by creating advanced *in vitro* models that replicate human organ structure and function 9, 16. These platforms incorporate microfluidics, biomaterials, and cell culture techniques to mimic dynamic organ microenvironments. Researchers can use them to explore organ physiology, disease mechanisms, and drug responses. In this section, we delve into various fabrication techniques for OOC systems, including microfluidics-based approaches, biomaterial-based strategies, and hybrid systems combining both methods. OOC systems aim to emulate key features of specific organs, employing controlled microfluidic environments to culture relevant cell types. They typically include microchannels, chambers, and porous membranes for cell culture, nutrient exchange, and simulating physiological fluid flow. Next, we discuss the integration of mini-tissues, produced using methods such as bioprinting, cell spheroids, microfluidics, and hybrid approaches, into OOC systems.

### Bioprinting-based OOCs

Recent advances in 3D bioprinting processes have enabled the recapitulation of *in vivo*-like conditions and the multiplex microarchitectures of human tissues. Therefore, bioprinting strategies are promising tools for designing and manufacturing OOC devices. In this regard, bioprinted OOCs can provide sophisticated structures using ECMs and cells with a fast-manufacturing process and easy modification of device design.

The integration of additional complexity through the incorporation of various cell types such as endothelial or nervous cells into the bioink of 3D bioprinting systems offers a notable advantage in generating biomimetic tissue constructs to create OOC models. This approach enables drug screening and disease modeling to represent native human tissues and disease responses more accurately.

For instance, Zhang et al. fabricated an endothelialized myocardium using 3D bioprinting of HUVECs, followed by seeding cardiomyocytes. The resulting organoid was placed within a perfusable microfluidic device, creating an “endothelialized-myocardium-on-a-chip” system for evaluating cardiovascular toxicity 71. Similarly, Lind et al. introduced a fully 3D-printed device capable of continuously detecting the contractile forces of cardiac tissues 72. This device incorporates multiple components, including a cantilever, strain sensor, and electrical interconnects. These results suggest that this device provides a novel platform for evaluating the morphogenesis and pathogenesis of drug-induced tissues through structural and functional assessments. In addition to the described applications, various examples of 3D bioprinting-based OOCs are presented in Table 2.

### Microfluidics-based OOCs

Microfluidics-based OOC platforms leverage microfabrication techniques to create precise and controllable fluid flow patterns, thereby enabling the emulation of organ-specific microenvironments. The design of microfluidic OOC devices involves integrating channels and chambers to mimic the vasculature and tissue architecture of the target organ 73. Microfluidic flow systems facilitate the transport of nutrients, oxygen, and other factors, thereby promoting cell viability and physiological functions. Additionally, microfluidic OOCs with multiple compartments can support crosstalk between various cells *via* the interchange of secreted factors. Shim et al. examined this phenomenon by designing microfluidic OOCs with two compartments containing tumor-draining lymph nodes and a tumor 74. The authors observed that the lymph nodes were significantly immunosuppressed when cultured with tumor cells compared with healthy tissues, suggesting that the designed microfluidic OOCs can recapitulate tumor-induced immune suppression in healthy lymph nodes.

Microfluidic systems allow the seeding of multiple cell types within a device, mimicking the cellular composition of the organ of interest. Additionally, these platforms enable the creation of barrier models, such as blood-brain or intestinal barriers, which are crucial for drug transport and disease modeling 75. Endothelial and epithelial cells create biological barriers between tissues. Griep et al. incorporated* the* blood-brain barrier by using a human brain endothelial cell line (hCMEC/D3) 76.

Barrier function was evaluated by analyzing barrier tightness using transendothelial electrical resistance measurements. As a result, the barrier functionality was enhanced when fluid shear stress was applied, whereas exposure to tumor necrosis factor inhibited this functionality. Moreover, Kim et al. incorporated the intestinal barrier using intestinal epithelial cells (Caco-2) cultured in microfluidic OOCs and exposed them to a cyclic mechanical strain similar to that in the native environment 77. As a result, mechanical stress can evoke the differentiation of Caco-2 cells, assist in villous structure formation, and perform intestinal barrier functions.

### Biomaterials in OOCs

Biomaterial-based OOC platforms employ biologically relevant scaffolds to support cell culture and tissue organization, thereby providing a more native microenvironment. A variety of natural biomaterials including collagen, alginate, gelatin, and decellularized extracellular matrix (dECM), and synthetic biomaterials including PDMS, PMMA, and PCL have been employed to serve as scaffolds in biomaterial-based OOC systems (Table 2). These materials offer structural support, cell adhesion sites, and signaling cues to guide cell behavior and tissue formation. In general, biomaterials with pH, oxygen, and carbon dioxide characteristics that are compatible with cell types should be considered. Previous studies have suggested that a balanced pH of approximately 7.4, and a carbon dioxide concentration of 4-10% are appropriate for supporting cellular functions 78, 79. In the context of tissue-specific biomaterials in OOC, the dECM, which consists of collagen, elastin, glycoproteins, and various tissue-specific growth factors, has been regularly employed to recapitulate the native environment of cells.

The mechanical properties of the biomaterial matrix are important criteria for the fabrication of OOCs 80. The integrins located on the membranes of cells reportedly can detect the mechanical properties of the substrates and relay these signals in multiplex intercellular pathways to control the biological functions 73, 81, 82. Generally, mechanical properties that resemble native tissues can evoke favorable cellular bioactivities. For example, the elastic modulus of an arterial wall is approximately 1 MPa, similar to that of PDMS (approximately 700 kPa) 83. Therefore, researchers used PDMS in OOCs to recapitulate their native mechanical properties. In addition, the stiffness of the lung tissue was measured as approximately 3.4 kPa 84. To obtain similar mechanical properties, Huang et al. examined Gel-Ma bioconstructs with a stiffness of 6.23 kPa 85.

The cells were seeded within the biomaterial scaffold, which allowed them to self-organize and differentiate into tissue-like structures. The 3D architecture of the scaffold facilitates cell-cell interactions and recapitulates tissue-specific functions 86. The framework of biomaterial-based scaffolds requires an interconnected pore network that allows the continuous flow of the medium 87. The exchange of nutrients and gases, as well as the removal of metabolic waste and byproducts from scaffold degradation, can assist in supporting cellular functions. However, the porosity of the 3D scaffold must be finely balanced so that the mechanical integrity and stability are not compromised. Based on previous literature, the selection of an appropriate biomaterial with a fine balance between mechanical properties, porosity, and bioactivity is a crucial factor to consider in the successful design of OOC systems.

### Hybrid OOC systems

The previously outlined fabrication techniques for Organ-on-Chip (OOC) platforms offer valuable tools for *in vitro* modeling of human organs. However, it's important to recognize that these methods come with both advantages and disadvantages. For instance, 3D bioprinting systems have notable strengths for OOC applications. They are compatible with a wide range of hydrogels, making them suitable for multi-material bioprinting. Additionally, they excel in achieving high manufacturing consistency, enabling the printing of solutions with high cell density. Nevertheless, it's crucial to consider their limitations. Hydrogels in this context can degrade rapidly, and the shear forces and pressures involved in extrusion may affect cell viability. Moreover, 3D bioprinting systems for OOCs may face restrictions in terms of printing speed and resolution. To address the challenges associated with bioprinting, precise fluid control in microfluidic-based approaches allows for the fabrication of mini-tissues with high resolution and cell viability. However, it's worth noting that the complex setup and potential absence of tissue-specific biochemical cues could potentially hinder the bioactivities of the residing cells. In light of this, biomaterial-based strategies may offer a more native-like microenvironment.

Currently, hybrid OOC systems that combine bioprinting, microfluidics, and biomaterials are being introduced to create more comprehensive and functional organ models. In hybrid systems, microfluidic channels and chambers are integrated into biomaterial-based scaffolds, enabling the precise control of fluid flow and cell culture conditions. This integration enhances the physiological relevance of the OOC model. For instance, cell spheroids have been intensively studied as they are relatively easy and inexpensive to form, while providing a valid evaluation to study multicellular interactions with external stimuli. Although spheroids have regularly been studied, their static culture environment can cause the accumulation of biochemical wastes and affect cell viability. To overcome these issues, cell spheroids have frequently been integrated into OOCs to evaluate drug efficacy and the influence of stimuli on cellular behavior. For instance, Alexander et al. fabricated 3 × 3 microwells using a commercially available 3D printer to place the HepG2 spheroids into each well 88. Subsequently, various parameters, including extracellular acidification, cellular respiration, and morphology, were monitored to assess the metabolic activity of the spheroids. However, they have several drawbacks, including fragility, limited vascularization, and limited dimensions and lifetimes 89.

Hybrid OOC systems represent a powerful tool in biomedical research, combining the adaptability of microfluidics with the biomimetic qualities of scaffold materials. These systems enable researchers to delve into the intricacies of cellular interactions and tissue behaviors in a controlled laboratory environment. However, it's worth noting that the integration of diverse components can present technical challenges, particularly in terms of both fabrication and precise fluidic control. Overcoming these hurdles is pivotal in realizing the full potential of these systems for advancing our understanding of biological processes.

Microfluidics-based approaches offer precise fluid control and cellular integration, whereas biomaterial-based strategies provide a more native-like microenvironment. Hybrid systems combine these technologies to create comprehensive and functional organ models. For instance, Urbaczek et al. investigated OOC integrated with microfluidic channels containing endothelial cells. To enhance the bioactivities of the endothelial cells, the channel was treated with oxygen plasma followed by fibronectin coating 90. As a result of these treatments, a notable increase in vascular endothelial growth factor (VEGF) secreted from the cells was observed. These OOC platforms have tremendous potential for advancing disease modeling, drug screening, and personalized medicine, contributing significantly to the fields of tissue engineering and regenerative medicine. In the following section, we explore the integration of mini-tissues or organoids with these OOC platforms to create tumor-on-a-chip models, presenting a powerful approach to cancer research and therapeutic development.

## Applications of tumor-on-a-chip (TOC) combined with mini-tissues/organoids

The integration of mini-tissues or organoids with advanced microfluidic platforms has provided exciting opportunities in cancer research, culminating in the development of TOC models. These innovative systems combine the physiological relevance of 3D mini-tissues with the dynamic microenvironments provided by microfluidics, enabling the recreation of the complex tumor microenvironments (TME) *in vitro* 40, 91, 92. In this section, we explore different approaches for creating TOC models using various biofabrication methods, highlighting their contributions to understanding tumor biology, drug responses, and personalized medicine.

### TOC with 3D bioprinted tissue constructs

**Figure 5:**
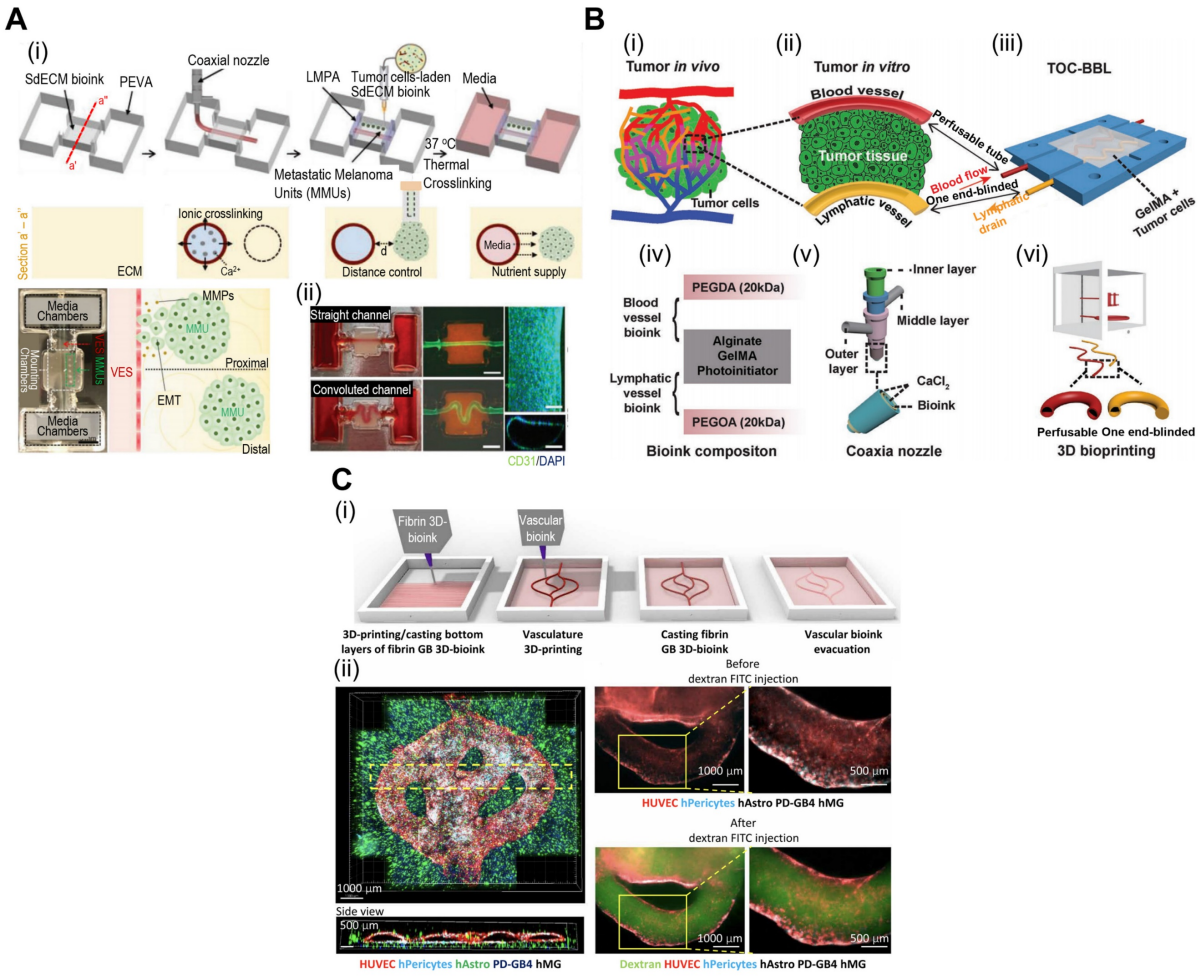
**Applications of 3D bioprinted tissue-constructs incorporated in TOC.** (**A**) (i) Schematic illustrations of the bioprinting process for the organ-on-a-chip (OOC), and (ii) optical images of the perfusion of OOC and flurescent images of DAPI (blue)/CD31 (green) demonstrating vascularization of perfuable OOC. Adapted with permission from 93, copyright Wiley 2021. (**B**) Schematic showing (i) native and (ii) designed lymphatic-blood system of tumor microenvironment. (iii-vi) Schematic diagram of the fabrication process of the OOC demontrating perfusable blood (red) and flymphatic (yellow) hollow tubes. Adapted with permission from 94, copyright Wiley 2019. (**C**) (i) schematical diagram illustrating 3D bioprinting of multistage microfluidic OOC and (ii) confocal imaging demonstrating HUVEC (red) and pericyte cells (blue) within the OOC. Adapted with permission from 95, copyright AAAS 2021.

The precise control of cell positioning and tissue architecture afforded by 3D bioprinting makes it an excellent choice for creating TOC models with mini-tissues or organoids. By bioprinting different cell types within mini-tissues, researchers can recreate the heterogeneity observed in tumors* in vivo*, including cancer, stromal, and immune cells. The spatial arrangement of these cells can mimic the tumor architecture and cellular interactions, enabling the study of tumor growth and progression. For instance, Kim et al. have demonstrated *in situ* bioprinting of metastatic cancer spheroids (500-1000 μm) to a perusable vascular system (Fig. 5A) 93. The authors used two cancer types [malignant melanoma (SK-MEL-28) and gastric carcinoma (HTB-103)]. The cancer spheroid with a diameter of approximately 600 μm showed optimal hypoxia, invasion, and angiogenic activities. Based on these results, the authors concluded that the developed platform can recapitulate patient-specific cancer progression. Similarly, Cao et al. incorporated perusable blood and lymphatic vessels around human breast cancer cells (HTB-22) to recreate the *in vivo* TME, as shown in Fig. 5B(i)-(iii) 94. They have investigated various combinations of blood and lymphatic vessels using polyethylene glycol diacrylate as the vessel wall to observe the diffusion profiles [Fig. 5B(iv)-(vi)]. As shown in Fig. 5C(i), the vascular network can also be incorporated into TOC *via* submerged bioprinting 95. Submerged bioprinting methods have regularly been utilized to support bioprinting and enhance resolution 23, 96. The research conducted by Neufeld et al. demonstrated the deposition of a thermoreversible synthetic polymer (Pluronic F127) as a sacrificial material to provide a vascular network for 3D bioprinted glioblastoma (the most aggressive form of brain cancer). Compared with the 2D cancer model, the dynamic microenvironment provided by the 3D bioprinted model can provide a more accurate and reliable evaluation of cancer progression and drug efficacy.

TOC models with 3D bioprinted mini-tissues offer a valuable platform for drug screening and personalized medical approaches. These models can be derived from patient-specific cells, allowing researchers to test the efficacy of various therapies and identify personalized treatment strategies. Moreover, a multi-barrel bioprinting strategy can provide an appropriate TME for the accurate evaluation of cancer therapeutics 97, 98. Yi et al. utilized a multi-barrel 3D bioprinting method for human glioblastoma cells in the core and endothelial cells in the peripheral layer to provide an oxygen gradient for the bioconstruct 99. To provide biochemical cues such as the TME, the research group utilized brain dECM. The authors evaluated patient-specific reactions to various cancer treatments, including chemoradiation and temozolomide. Because of patient-specific compartments, including the core (glioblastoma) and peripheral (vascular) compartments, the reaction to treatment can provide personalized treatment. Bioprinted TOC, while a promising tool in cancer research, does have a significant drawback of relatively weak cell-cell interactions 100, 101. This may result in difficulties in accurately replicating the TME, potentially leading to simplified representations. Additionally, bioprinted TOC faces challenges in emulating the dynamic nature of tumors and their responses to treatment over time. To address these issues, cell-spheroid based TOC address the formal challenges *via* providing robust cell-cell interactions, while microfluidic-based TOC can overcome the latter challenge *via* providing dynamic culture environment to better mimic the native TME. In addition to the applications of bioprinted TOC, various examples are listed in Table 3.

### Spheroid-based TOC models

**Figure 6:**
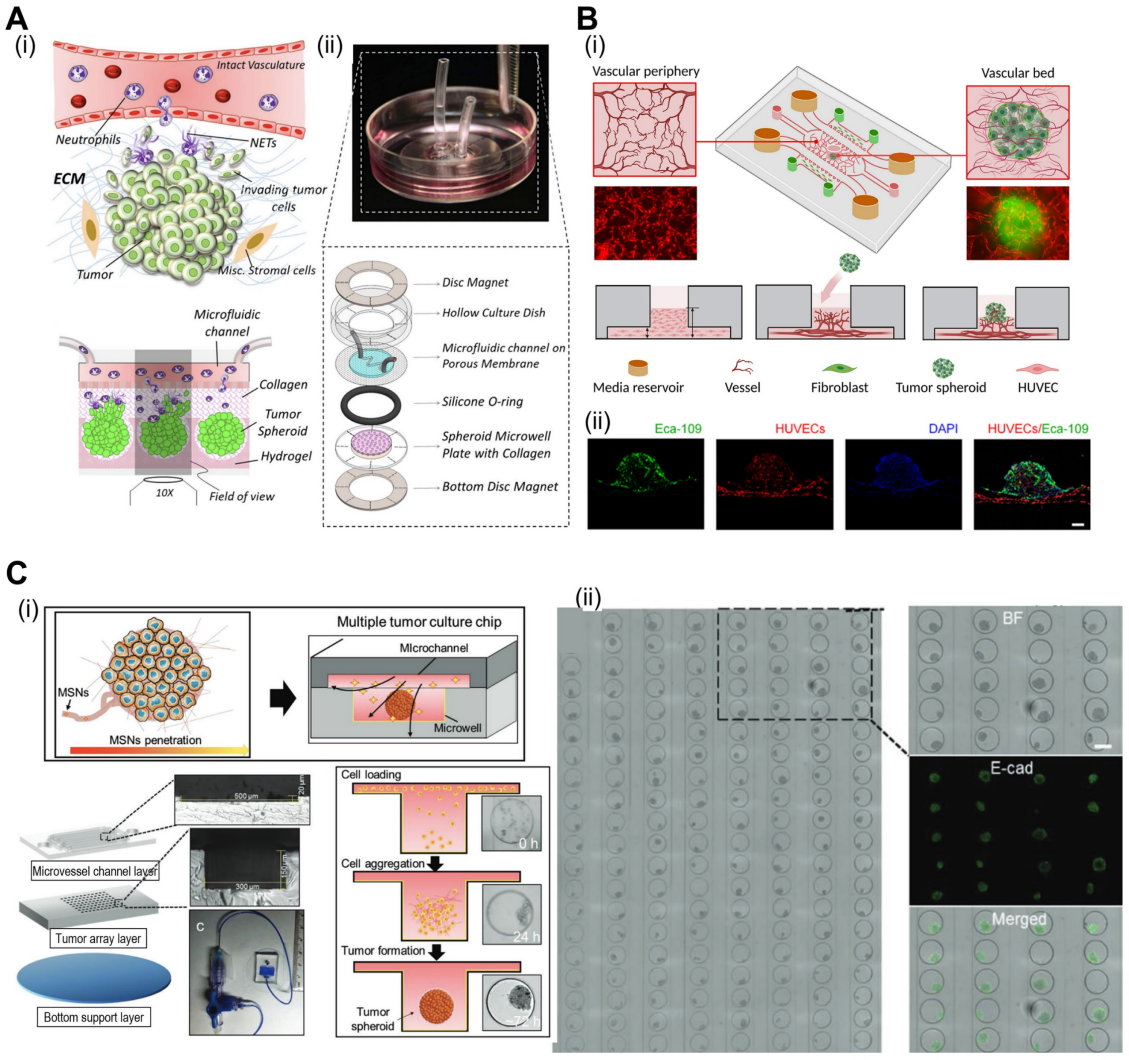
**Cell-spheroids incorporated in TOC applications.** (**A**) (i) Tumor microenvironment (TME) schematic illustration between interactional dynamics of pre-metastatic tumor and neutrophils and (ii) optical images demonstrating tumor spheroids incased within microwells. Adapted with permission from 103, copyright IOP publishing 2021. (**B**) (i) Schematic illustration of vascularized tumor spheroid organ-on-a-chip (OOC) comprised of five microchannels and (ii) fluorescence images of Eca-109 and HUVEC cells demonstrating vascularized tumor spheroids. Adapted with permission 104, copyright ACS publishing 2022. (**C**) Schematics and optical images of microfluidic OOCs containing MCF-7 cells demonstrating evaluation of nanoparticle penetration, and (ii) bright-field and fluorescent images of spheroid arrays containing 160 MCF-7 tumor spheroids. Adapted with permission 107, copyright Wiley 2019.

Spheroids serve as building blocks for TOC models, enabling the formation of mini-tissues that recapitulate aspects of TME gradients and cell-cell interactions. Spheroid-based TOC models enable the creation of nutrient and oxygen gradients, simulating the heterogeneous microenvironments observed in solid tumors. These gradients can influence tumor growth, metabolism, and drug responses, providing valuable insights into tumor behavior. As shown in illustrations of Fig. 6A(i), neutrophils can be extravasated through a phenomenon known as chemotaxis in response to tumor-derived stimuli 102. However, interactions between cancer cells and neutrophils are not fully understood. To evaluate these dynamics, Surendran et al. used spheroids composed of ovarian cancer cells and placed them in microwell plates coated with collagen [Fig. 6A(ii)] 103. Neutrophil migration and tumor invasion can be stimulated by incorporating microfluidic channels into microwell plates, which act as a vascular network. In the described work, the researchers noted elevated migration of neutrophils towards the cancer spheroids and the formation of neutrophil extracellular traps (NETs). Subsequently, the buildup of NETs can induce aggregated cancer cells in the spheroids to migrate and invade the surrounding collagen matrix.

Vascularized tumors are essential criteria for providing an appropriate TME and oxygen gradient for evaluating cancer therapies. Hu et al. developed tumor spheroids composed of human esophageal carcinoma (ECA 109) cells using microfluidic methods 104. Subsequently, these spheroids were placed in a vascular bed composed of HUVEC to induce vascularization [Fig. 6B(i)]. As shown in Fig. 6B(ii), vascular networks formed around the cancer spheroids. Previous studies have shown, that prolyl hydroxyl inhibitors can normalize tumor blood vessels, thereby improving cancer treatment efficacy 105, 106. Using the described TOC, researchers have evaluated the effects of dimethylallyl glycine (DMOG, a prolyl hydroxyl inhibitor). DMOG significantly improved the vascularization of cancer spheroids and the efficacy of cancer treatments (paclitaxel and cisplatin).

With the ability to create complex 3D structures, spheroid-based TOC platforms offer an opportunity to study tumor invasion and metastasis. The integration of microfluidics enables the observation of tumor cell migration and dissemination in real time, contributing to a better understanding of cancer metastatic processes. Moreover, microfluidics-incorporated TOC with tumor spheroids can be utilized for appropriate drug delivery. For instance, as shown in Fig. 6C, Zhuang et al. evaluated the effects of mesoporous silica nanoparticles (MSNs) on an array of breast cancer spheroids 107. Because of their biodegradability and biocompatibility, MSNs have been regularly used as nano-delivery systems for cancer therapy 108, 109. As such, the research group evaluated various conditions under which MSNs were administered to cancer spheroids. The group noted that continuous MSNs delivery resulted in better infiltration, and the size of the MSNs had a greater effect than the static 2D model. Additional examples of microfluidic TOCs are listed in Table 4.

The utilization of cell spheroids in Tumor-on-Chip (TOC) technology presents several notable limitations 110. Firstly, cell spheroids have inherent size restrictions, which may hinder the accurate representation of complex tumor structures and the heterogeneity typically observed in real tumors. Additionally, replicating the intricate vascular networks present in tumors proves challenging with cell spheroids alone, impacting the representation of oxygen gradients within the tumor. Further, the static culture conditions commonly used for spheroids do not adequately capture the dynamic nature of tumor growth and responses to treatment over time. To overcome these challenges, integration of complex vascular networks can inherently increase size of tumor spheroids while maintaining the oxygen gradient. Moreover, introduction of microfluidics can provide dynamic culturing conditions that recapitulate the native TME.

### Microfluidic TOC models

**Figure 7:**
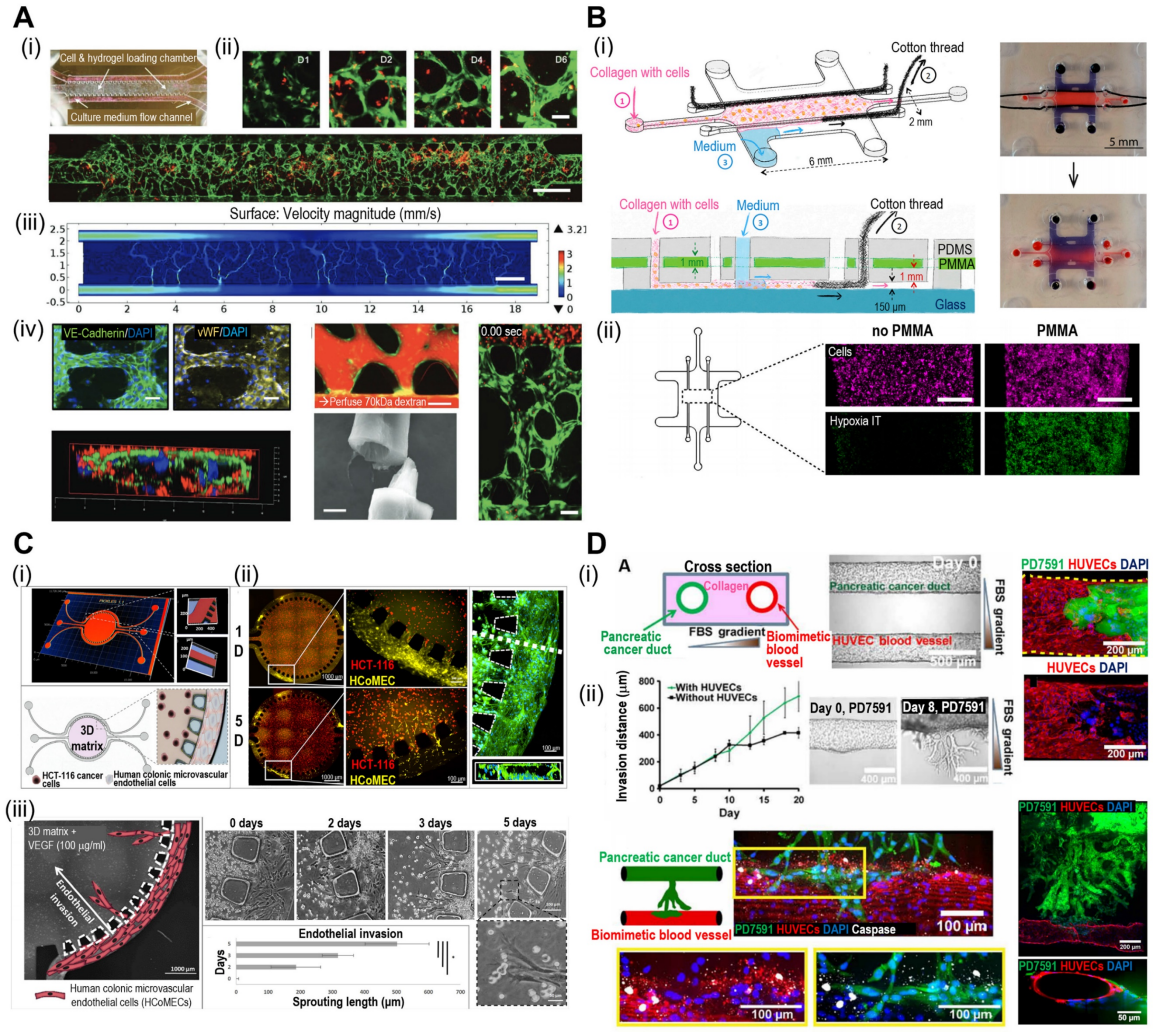
**Microfluidic-based TOCs.** (**A**) (i) Normal optical and (ii) fluorescent images showing live (green)/dead (red) microvasculature-based organ-on-a-chip OOC containing brain tumor stem-like cells. (iii) Finite element analysis (FEA) results of flow velocity profile measured using COMSOL software and (iv) immunofluorescent images (VE-cadherin and vWF), cross-sectional images of the microvessels. Adapted with permission 114, copyright Wiley 2019. (**B**) (i) Schematic diagram and optical images of OOC consisting of a central culture compartment and perfusion mechanism (blue: trypan blue). (ii) Evaluation of hypoxia of a PMMA-incorporated OOC. Adapted with permission 115, copyright MDPI 2019. (**C**) (i) A 3D heatmap and design of an OOC, (ii) fluorescent images demonstrating prevascularization of OOC, and (iii) schematic and optical images of endothelial cell sprout formations. Adapted with permission 116 copyright AAAS 2019. (**D**) (i) Schematic illustration and optical images of OOC consisting of blood vessels and pancreatic cancer ducts, and distance of pancreatic ductal adenocarcinoma cell invasion distance. (ii) Fluorescent images of YFP PD7591 cells invading the blood vessel. Adapted with permission 117 copyright AAAS 2019.

Microfluidic systems provide dynamic and controlled microenvironments for TOC models, allowing researchers to study tumor behavior under conditions that mimic the *in vivo* environment. Microfluidic TOC models can mimic fluid flow and shear stress in the TME. These dynamic conditions influence tumor cell behavior, including proliferation, invasion, and response to therapies, thereby providing critical insights into cancer biology. For instance, the perivascular niche (PVN) plays an important role in the intercellular activities of cancer cells, including cell fate, tumor invasion, and drug resistance 111, 112. In the context of brain cancer, the PVN refers to the region around the micro-vessels in which brain tumor stem-like cells (BTSC) reside, thereby providing a path for the migration of cancerous cells 113. Xiao et al. utilized TOC incorporated into the microvascular track to evaluate the role of the PVN in brain tumor stem-like cells (BTSC), as shown in Fig. 7A(i) 114. As shown in Fig. 7A(ii), the endothelial cells formed extensive vascular networks after 4 d of culture. To further elucidate the velocity profile, computational fluid dynamics (CFD) was conducted to determine the local flow rates, revealing heterogeneity within the microvessels [Fig. 7A(iii)]. Immunofluorescence images of VE-cadherin and vWF revealed the formation of cell-adherent junctions [Fig. 7A(iv)].

The oxygen gradient is an essential part of the TME and promotes tumor heterogeneity (hypoxic core and normoxic surroundings). The microfluidic device was integrated into the TOC to maintain an oxygen gradient that mimicked the native TME. Palacio-Castañeda et al. fabricated TOC-containing U-251 MG glioblastoma cells (Fig. 7B) 115. As a result, the research group indicated that under hypoxic conditions, the metabolic activities of cancerous cells increased, as indicated by higher glucose uptake and hydrogel acidity.

Microfluidic systems enable the real-time monitoring of cellular behavior and responses to various stimuli, such as drug treatments or changes in nutrient availability. This capability allows researchers to study the dynamic and temporal aspects of tumor responses, further enhancing our understanding of cancer progression and therapeutic outcomes. For instance, Carvalho et al. developed a 3D TOC integrated with microfluidic channels that simulated the TME of human colorectal tumors to evaluate anticancer drugs 116. Fig. 7C(i) and (ii) show the 3D surface mapping of the TOC and cell tracker of a human colorectal carcinoma cell line (HCT-116 CRC; red) and human colonic microvascular endothelial cells (yellow), respectively. Inside the circular core compartment, a 3D matrix composed of Matrigel and VEGF facilitated endothelial cell invasion [Fig. 7C(iii)]. The developed TOC allowed for the evaluation of real-time drug delivery and its cellular effects. Similarly, Nguyen et al. developed a TOC consisting of two ducts (pancreatic cancer and vascular ducts) separated by a collagen matrix, as shown in Fig. 7D(i) 117. Cancerous cells (pancreatic ductal adenocarcinoma) invaded the vascular network, resulting in endothelial ablation [Fig 7D(ii)]. Additional examples of microfluidic TOCs are listed in Table 4.

Microfluidic-based TOC platforms, while immensely powerful for emulating the intricacies of the tumor microenvironment, face a notable challenge in scalability 118. The intricate networks of microchannels and chambers that define these systems can be complex and time-consuming to fabricate, limiting the ease with which they can be scaled up to accommodate larger-scale experiments or high-throughput screening. To address the scalability challenge inherent in microfluidic-based Tumor-on-Chip (TOC) systems, a multifaceted approach can be employed. Recent advanced fabrication techniques, such as 3D printing and soft lithography, offer a scalable means to create intricate microfluidic structures efficiently. Additionally, Modular design principles facilitate scalability by allowing the assembly of smaller, standardized components into larger, more complex systems. By integrating these strategies, the scalability issue can be effectively addressed, unlocking the full potential of microfluidic-based TOC systems for advancing cancer research and drug development.

TOC technology, while a promising tool in cancer research, does come with inherent limitations. These models are simplified representations of real tumors, often lacking the full complexity and heterogeneity found in the human body. They may focus on specific aspects of tumor biology, potentially missing critical interactions. Furthermore, the absence of immune system representation and challenges in modeling metastasis are notable drawbacks. Size restrictions, maintaining long-term viability, and ethical considerations also pose challenges. Additionally, the resource-intensive nature and potential variations between models can impede widespread adoption. Integrating systemic effects and validating against clinical data remain ongoing concerns. Despite these limitations, TOC models have significantly advanced our understanding of cancer, though ongoing research seeks to address and overcome these constraints for even greater efficacy.

## Future perspectives

TOC technology, which combines mini-tissues or organoids with advanced microfluidic systems, has been accompanied by a new era of cancer research and therapeutic development. The integration of various biofabrication methods into TOC models offers several applications and holds significant promise for personalized medicine, high-throughput drug screening, and advancements in cancer biology. In this section, we explore the various applications of TOC technology and discuss future perspectives that could shape its impact on cancer research and healthcare.

TOC models provide a physiologically relevant representation of the tumor microenvironment, allowing researchers to study tumor growth, invasion, metastasis, and therapeutic responses in a controlled and reproducible manner 119. These models can bridge the gap between traditional 2D cell culture and *in vivo* studies, enabling more accurate and predictive preclinical testing of drugs and therapeutic strategies. The ability to incorporate different cell types, such as cancer, stromal, immune, and vascular cells, into mini-tissues or organoids adds to the complexity of the model and better reflects tumor heterogeneity *in vivo*. This facilitates the investigation of tumor-stromal interactions, immune responses, and the effects of the TME on therapeutic efficacy.

Although the TOC technology shows great promise, there are still challenges to be addressed in terms of scaling and standardization. The complexity of these models and the need for specialized equipment may limit their accessibility. To fully utilize the potential of TOC technology, efforts should be made to optimize and standardize fabrication processes, making them more accessible and reproducible.

The integration of TOC technology with other cutting-edge biotechnologies such as artificial intelligence (AI) and high-throughput screening has opened new avenues for cancer research and drug development. AI algorithms can analyze the complex data generated by these models and provide valuable insights into tumor behavior, drug responses, and potential therapeutic targets. High-throughput screening platforms can rapidly test many drugs or combinations, thereby accelerating the identification of novel anticancer agents and personalized treatment regimens.

TOC technology can revolutionize personalized medical approaches. Using patient-derived cells to create mini-tissues or organoids on chips, researchers can tailor treatments for individual patients, leading to more effective and targeted therapies. These personalized models can be used to predict drug responses and optimize treatment strategies to achieve better clinical outcomes. Furthermore, TOC platforms can be instrumental in testing novel drug candidates, evaluating drug combinations, and screening for potential toxicity, thereby expediting drug development and reducing reliance on animal testing.

The future of TOC technology is expected to be positive, with many exciting directions and emerging technologies. Advances in biofabrication techniques such as improved 3D bioprinting strategies, novel biomaterials, and more sophisticated microfluidic platforms will enhance the capabilities and versatility of TOC models. The incorporation of immune cells and patient-specific immune responses adds another layer of complexity to these models, facilitating the study of cancer immunotherapy and immunomodulatory drugs. Furthermore, the integration of OOC systems representing different organs and tissues into a single “body-on-a-chip” platform may enable comprehensive studies of systemic drug effects and organ crosstalk.

## Conclusion

TOC technology represents a promising frontier in cancer research, offering sophisticated and physiologically relevant models with the potential to reshape the landscapes of cancer treatment and care. Integrating biofabrication methods, mini-tissues, and microfluidics offers a powerful toolkit for understanding the complexities of tumor biology and personalized medicine, bringing us closer to more effective and targeted cancer therapies. With continued research, innovation, and collaboration, TOC technology holds tremendous promise for shaping the fight against cancer and advancing the boundaries of biomedical science.

## Figures and Tables

**Table 1:** Fabrication methods of 3D mini-tissues/organoids.

Fabrication type	Method	Material	Cell	Results	Ref.
**3D Bioprinting**	Printing transwell dermis, vascular channel, hypodermis, epidermis	Hypodermis: adipose-derived dECM, fibrinogen Dermis: skin-derived dECM, fibrinogen Vascular channels: gelatin, glycerol, thrombin Transwell: PCL	HPAD HUVEC HDF HEK	Increased structural complexity Fully-matured perfusable vascularized 3D skin models were obtained Structural similarity compared with the native human skin using skin stemness markers	120
	Printing bioink into the supportive bath followed by a crosslinking process	Bioink: alginate, carboxymethyl chitosan Printing bath: PEI	HAT-7	Highly porous structure with sufficient structural integrity was obtained High degree of printability Mineral deposition and enamel-like tissue formation	121
	Bioink were crosslinked within the glass capillary nozzle and wall shear stress induced cell alignment	Gel-Ma	ASC	Significant reduction in the extrudate swelling phenomenon Higher cellular alignment and MHC expression Improved muscle regeneration	122
	Fibroblast and keratinocyte layers printed using fish derived bioink and UV crosslinking process	Tilapia skin derived dECM-Ma Cod skin derived dECM	HS27 HaCaT	Enhanced *in vitro* cellular activities Enhanced mechanical properties	123
**Spheroids**	Positioning spheroids within the self-healing support hydrogel	Supportive hydrogel: HA modified with adamantane or β-cyclodextrin	iPSC-derived cardiomyocytes	3D bioprinting of high cell density tissue models, with precise control over microtissue structure and local heterogeneity	124
	Electrospinning spheroid-laden bioink on PCL strut	Bioink: alginate/PEO Supportive strut: PCL	C2C12	Uniaxially elongated spheroids Spheroid-laden structure greatly promoted myotube alignment, fusion, and maturation. Upregulated myogenic-related gene (MyoD1, myog, and Myh2) expression	50
	Providing a rotational motion to the hanging droplets to facilitate the aggregation of suspended cells	N/A	HSF HepG2	Overcome current challenges in the cell spheroid production process including long preparation time, low cell viability, and high costs	125
	Using magnetic field to form spheroids with magnetic nanoparticles containing cells	Nanoparticles isolated from magnetic bacteria	BMSC AMB-1	Reduced the time required for spheroid generation Reinforced cell-cell contact for the activation of cellular interactions due to the magnetic forces Improve the various cellular functions such as proliferation and differentiation	126
	Using PCL-based buckyballs as micro-scaffolds for fabricating spheroids	PCL	ASC	Enhanced cell retention, the decreased compaction and the better control over the size Better chondrogenic and osteogenic differentiation	127
**Microfluidic systems**	Cell laden bioink was extruded into pro-printed microchamber	Microchamber: PCL Bioink: Gel-Ma	MSC	Printing scalable arrays of spheroids within a reinforcing polymeric framework to orientate spheroid growth and fusion Supporting endochondral bone formation and maintaining the entire construct in bioreactor culture to enhance tissue development	128
	3D printing bioink containing cell-laden microgels which fabricated by microfluidic systems	Microgel: collagen/alginate Bioink: Sil-Ma, Gel-Ma	BMSC	Improved cell proliferation Better bone formation performance compared with 15%Sil-Ma/Gel-Ma construct	62
	Using multi-barrel capillary for fabricating complex microfibers	Alginate ECM protein	HepG2 NIH 3T3 HUVEC	Fabrication of bioactive microfibers with tunable morphological and structural features	63
	Epoxy resin-based T-junction microfluidic device for fabricating cell aggregated structures during printing process	Bioink core: Gel-Ma Bioink shell: collagen / PEGDA	NSC-34 C2C12	Promotion of neurogenic activity Exhibited significantly higher myogenic and neurogenic differentiation Higher NMJ formation than conventional bioconstructs	61
	PDMS based microfluidic device for fabricating cell-imprinted substrate and dynamic cell culturing	Chip: PDMS	ASC Chondrocyte	Regular patterning of cell membranes, which increased the efficiency of culture compared to previous irregular methods Cell-imprinted substrate using a microfluidic chip derived from the cell membrane pattern can be used for various cell culture applications	129
**Hybrid fabrication methods**	Using microfluidic device for formation cell-laden microgels	Thiolated gelatin Vinyl sulfonated hyaluronic acid	BMSC	Injected into defect site without sacrificing the cell viability and self-assemble into cartilage-like tissue via cell-cell interconnectivity Facilitate the cartilage repair	130
	4 × 10^5^ cells were plated into one well of ultralow attachment 6-well plate for cell aggregation to self-assemble into vascular organoids	N/A	ESC-derived EVC	More accessible for complex vasculature engineering Engineered cardiac tissues with high-density microvasculature composed of hESCs-derived EVCs	131
	Using microfluidic device for generation of double emulsion droplets and formation cell-laden multicompartmental microgels by self-assembly	Alginate	MCF-7 MDA-MB-231	Simple, affordable, and high-throughput approach for fabrication of complex microparticles Method offers co-encapsulation of viable cells with high resolution	132
	Loading MSC-laden micro-niches in meshed frames to induce self-assembly	Gelatin	Human umbilical cord MSC	Superior ability to maintain phenotypic characteristics and stemness in MSCs, while suppressing senescence and enhancing their paracrine functions Successfully repaired articular cartilage defects	133
	Cell-laden bioink extruded in cell-laden beads which fabricated by microfluidic	Collagen Gel-Ma Fibrin	BMSC iPSC-EC	Feasibility of 3D printing vasculature inside a bath of cell-laden microbeads to create centimeter-sized modular tissue engineered constructs	134

**Abbreviations:** PCL (poly(ε-caprolactone)); PEO (poly(ethylene glycol)); Gel-Ma (methacrylated Gelatin); PEGDA (poly(ethylene glycol diacrylate)); dECM (decellularized extracellular matrix); HPAD (human preadipocyte); HUVEC (human umbilical vein endothelial cells); HDF (human dermal fibroblast); HAT-7 (dental epithelial cell line); ASC (adipose stem cells); HS27 (fibroblast cell line); HaCaT (keratinocyte); HEK (human embryonic kidney cell); HSF (human splenic fibroblast); HepG2 (human liver cancer cell line); MSC (mesenchymal stem cells); iPSC (induced pluripotent stem cells); hESCs (human embryonic stem cells); MyoD1 (myogenic differentiation 1); Myog (myogenin); Myh2 (myosin heavy chain 2).

**Table 2:** Biomaterials for organ-on-a-chip (OOC).

Type	Material	Advantages	Disadvantages	OOC applications	Ref.
**Natural**	Collagen	Biocompatible Biodegradation High cell adhesion Appropriate permeability Main component of native tissue	Insufficient mechanical stability High cost of production	Lung Liver Intestine Brain	135-138
	Chitosan	Biocompatible Biodegradation	Unstable mechanical properties	Barrier model Gut	139, 140
	Alginate	Low cost of production Biocompatible Biodegradation Controllable mechanical properties Appropriate permeability	Inadequate cell adhesion Poor bioactivities Lack mammalian representation	Liver Vessel	141, 142
	Cellulose	High mechanical strength Insoluble in water Biocompatible	Lack mammalian representation Microbial and fungal degradation	Barrier model Liver	143, 144
	Gelatin	Low cost of production Biocompatible Biodegradation Appropriate permeability	Insufficient mechanical stability Temperature sensitive	Cardiac Liver Neuromuscular	145-147
	Matrigel	High cell adhesion Similar biochemical cues to the native tissue Appropriate permeability	High cost of production High variability from batch to batch Insufficient mechanical stability	Pancreas Brain Vascular	148-150
	dECM	Accurate representation of the native tissue High cellular activities Biocompatible Biodegradable Appropriate permeability	High cost of production High variability from batch to batch Insufficient mechanical stability	Multi-OOC	151
**Synthetic**	PCL	Low cost of production Biocompatible Sufficient mechanical properties	Low cell adhesion and activities Slow degradation	Supportive structure to bioconstructs	78
	PDMS	Optical transparent Highly elastic Biocompatible Can withstand long cyclic loading durations Low cost of production	Non-biodegradable Hydrophobic Low permeability	External OOC structure	51, 152
	PMMA	High mechanical properties Optical transparent Biocompatible Low cost of production	Non-biodegradable Hydrophobic	External OOC structure	153, 154
	PLA	Biodegradable Tunable mechanical properties Biocompatible	High stiffness not suitable for OOC Hydrophobic	External OOC structure Supportive structure to bioconstructs Membrane structure	155, 156
	PGA	Biodegradable Biocompatible	High stiffness not suitable for OOC Hydrophobic	Controlled drug release in OOC Membrane structure	78, 157

**Abbreviations:** PDMS (poly(dimethylsiloxane)); PMMA (poly(methyl methacrylate)); PLA (poly(lactic acid)); PGA (poly(glycolic acid))

**Table 3:** Fabrication techniques for organ-on-a-chip (OOC).

Type	Method	Material	Cell	Results	Ref.
**3D printing based OOC**	By printing OOC and embedded sensor	Ink for OOC: dextran, TPU, CB, PDMS, Ag:PA	hiPS-CMs NRVMs	3D printed cardiac microphysiology device fabricated by sequentially printing the OOC base, tissue template, and well	72
	By printing tissues and chip at a once	Ink for OOC: PCL Ink for tissue: type I collagen, gelatin	HUVEC HepG2	Liver-on-a-chip fabricated *via* simultaneously printing cavity, the cell laden hydrogel and cover Heterotypic cell types and biomaterials were successfully positioned at the desired position	158
	By printing microchannel within the PDMS bath	Ink for channel: carbopol Ink for OOC: PDMS	BMEC HUVEC	OOC with microchannel was developed by printing sacrificial carbopol within the PDMS bath	159
	By printing silicone bioink	Ink for OOC: silicone Ink for the tissue: brain dECM	U-87 MG HUVECs	Chamber of the vascularized GBM-on-a-chip was fabricated by printing permeable silicone-based ink Bioprinted glioblastoma-on-a-chip reproduces the results of patient-specific resistances to treatment	99
**Microfluidic based OOC**	Molding	Chip: PDMS, PTFE tube, PMMA Tissue: patient derived tissue slice	TDLN NDLN	Chip is composed of three-layer which containing reservoir, tissue culture well, PCL membrane Co-culture of pairs of tissue slices under continuous recirculating flow resulting in modeling of complex inter-organ communication *ex vivo*	74
	Laser cutting technique	Chip: PMMA Hydrogel for fluid: matrigel	hiPSCs-F	Custom and reconfigurable assembled chip composed of microfluidic chip and a fluidic routing unit is developed Various chip assemblies demonstrate the versatile utility of the fluidic routing system which enables custom design of the chip-to-chip communication and fitting of variety of multicell biological models	149
	Molding	PDMS	HepG2 hiPSC-HC	Integrated network of liver-lobule-like hexagonal tissue-culture chambers constructed in a hybrid layout with a separate seed-feed network integrated to chip Provides a microphysiological niche for hepatocytes	160

**Abbreviations:** TPU (thermoplastic polyurethane); CB (carbon black nanoparticles); Ag:PA (silver particle-filled, polyamide); GBM (glioblastoma); PTFE (Poly(tetrafluoroethylene)); hiPSC-CMs (Human-induced pluripotent stem cell-derived cardiomyocytes); NRVMs (Neonatal rat ventricular myocytes); HAT-7 (dental epithelial cell line); HepG2 (human hepatocellular carcinoma); HUVEC (human umbilical vein endothelial cells); BMEC (human bone marrow microvascular endothelial cell line); U-87 MG (GBM-like cell line); TDLN (tumor-draining lymph nodes); NDLN (non-draining lymph nodes); hiPSCs-F (human induced pluripotent stem cells-derived skin fibroblasts); hiPSC-HC (human induced pluripotent stem cells- derived hepatocytes).

**Table 4:** Applications of tumor-on-a-chip (TOC) combined with mini-tissues/organoids.

Type	Method	Material	Cells	Results	Ref.
**Bioprinted tissue constructs-based TOC**	3D bioprinted vessel, tumor within the dECM bath	Bioink for tumor: skin dECM Bioink for vessel: vascular tissue dECM, alginate Chip: PDMS	HTB-103 SK-MEL-28 A548 U-87 HUVEC	Metastatic cancer unit (MCU) shows hypoxia, invasion, and angiogenetic signaling Observed the proximity of MCU to vascular endothelium system (VES) augments the epithelial-mesenchymal transition (EMT) in MCU and vascular Dysfunction/inflammation in VES	93
	3D bioprinted cell laden perfusable hollow vessel and single oulet lymphatic vessel	Bioink for vessel: Gel-Ma, alginate, PEGDA Bioink for lymphatic vessel: alginate, Gel-Ma, PEGOA Chip: PDMS, PMMA	MCF-7 HUVEC	Systems with the bioprinted blood/lymphatic vessels exhibit varying levels of diffusion profiles for biomolecules and anticancer drugs	94
	3D bioprinted GBM and vessel	Bioink for GBM: fibrin/gelatin Bioink for vessel: PF-127 Chip: PDMS	U-87 MG T98G U373 293T cells Saos-2 MDAMB-231 GL261 HUVEC Vascular brain pericytes PD-GB4 cell	Bioprinted human and murine GB model was able to recapitulate fundamental aspects of *in vivo* GB models, including cell proliferation, invasion, response to therapies, and gene profiling	95
	3D bioprinted neuroblastoma and vessel, endothelial cells seeded vascular channel	Bioink: Gel-Ma, fibrinogen Chip: PMMA	HDMEC STA-NB15 ASC iPSc HUVEC/hTERT-EYFP HFF/hTERT-ECFP	Micro-vascularized neuroblastoma tumor-environment model directly printed into fluidic chips and observed the interaction between vascular system and neuroblastoma tumor	161
**Spheroid-based TOC models**	Spheroid was placed within the chip	Chip: type-I bovine collagen, PAAm ring magnet	NIH OVCAR-3	Distinct neutrophil responses and functional modalities respond to the growing tumor spheroids	103
	Spheroid generated within the chip	Chip: PDMS (coated with PVA)	HCT11 T47D HepG2	Tumor spheroids were generated within the chip and used to evaluate drug efficiency (anticancer drug efficacy, apoptosis) Results indicate negative correlation between the dimension of tumor spheroids and drug sensitivity	162
	Spheroids was placed within the vascularized chip	Chip: PDMS	HUVECs hLFs Eca-109	Perfusable, vascularized tumor spheroid-on-a-chip was used to observe the vasoprotective and angiogenic effects of the drugs	104
**Microfluidic-based TOC models**	Cell suspension was flowed into the microfluidic chamber	Cell suspension: fibrinogen, thrombin Chip: PDMS	ECFC-EC NHLF SW480 HCT116	Colorectal cancer (CRC) confirmed using gene expression, and tumor heterogeneity Treatment responses in the bioconstructs closely recreate the CRC tumor clinicopathology	163
	Cell suspension was injected into the microfluidic chip	Cell suspension: fibrin, thrombin Chip: PDMS	HUVEC GS5	Microvasculature-on-a-chip system model can recapitulate *in vivo* tumor cell dynamics and heterogeneity, representing a new route to study patient-specific tumor cell functions	114

**Abbreviations:** PVA (Poly(vinyl alcohol)), HTB-103 (gastric carcinoma cells); SK-MEL-28 (Malignant melanoma cell); A548 (lung carcinoma cell); U-87 (glioblastoma); MCF-7 (breast cancer cell line); A-172 (Human Glioblastoma cells); hCMEC/D3 cerebral micro-vessel endothelial cells (hCMEC/D3); GBM (Glioblastoma multiform); T98G, U373, U-87 MG (human glioblastoma cell lines); Saos-2 (human osteosarcoma cells); MDAMB-231 (human breast cancer cell); GL261 (murine glioma cell line); STA-NB15 (neuroblastoma cell line); ASC (adipose derived stem cell); HFF/hTERT-ECFP (human foreskin fibroblasts); NK-92 (cellosaurus cell line); ECFC-EC (Human endothelial colony-forming cell-derived endothelial cells); NHLF (Normal human lung fibroblasts); SW480 (colorectal cancer cells); HCT116 (colorectal cancer cell); NIH:OVCAR-3 (Chemoresistant ovarian cancer cell line); PAAm (poly(acrylamide)); T47D (human breast cancer cell line); hLFs (normal human lung fibroblasts); Eca-109 (human esophageal carcinoma cell line); GS5 (patient-derived glioma stem-like cells).
